# Incidence of Oropharyngeal Cancer Among U.S. Active Component Service Members, 2005–2024

**Published:** 2026-05-15

**Authors:** Shauna L. Stahlman, Erika Dreyer

**Affiliations:** Epidemiology and Analysis Branch, Armed Forces Health Surveillance Division, Public Health Directorate, Defense Health Agency, Silver Spring, MD: Dr. Stahlman, Ms. Dreyer

Oropharyngeal cancer develops in the oropharynx, which is comprised of the soft palate, side and back walls of the throat, tonsils, and back of the tongue. ^1^ Oropharyngeal cancer is distinguished from cancers arising in the oral cavity and pharynx, otherwise known as head and neck cancers, which include the lip, salivary glands, mouth and gums, and entire throat (or pharynx) and tongue. ^2^

Oropharyngeal cancer comprises 2 distinct cancers, HPV-positive and HPV-negative types, with different risk factors and age distributions. It is estimated that 60-70% of oropharyngeal cancers in the U.S. are due to infections with high-risk types of human papillomavirus (HPV), with smoking and heavy alcohol use acting as important risk factors for HPV-negative types. ^3^ HPV-positive cancers tend to be diagnosed in people younger than age 50 years, whereas HPV-negative types tend to be diagnosed among older individuals. ^4^ In addition, HPV-positive oropharyngeal cancers tend to have better prognosis and respond better to treatment. ^5^ The first HPV vaccine became available in the U.S. for women ages 9-26 years in 2006. ^6^ A bivalent vaccine became available in 2009, and a 9-valent vaccine became available for women and men in 2014. ^6^

Despite the availability of the HPV vaccine over the past 20 years, data published in 2025 indicate that incidence of oropharyngeal cancer in the U.S. increased slightly between 2006 and 2022, primarily among men and older individuals. ^7^ A previous study by the Murtha Cancer Center compared incidence rates of oral cavity and oropharyngeal cancers among active duty service men and men in the U.S. population between 1990 and 2013. ^8^ That study found that active duty incidence rates of oropharyngeal cancer were higher than U.S. population rates among non-Hispanic White individuals (IRR 1.19, 95% CI 1.01, 1.39) and men ages 40-59 years (IRR 1.18, 95% CI 1.00, 1.39), and rates increased for both populations over time. ^8^

Continued oropharyngeal cancer surveillance among U.S. service members was identified as a gap by the DHA public health cancer surveillance community of interest, and a new surveillance case definition for oropharyngeal cancer was created. This analysis represents the first use of the new case definition. This study aimed to examine the trend in annual incidence of oropharyngeal cancer among U.S. active component service members (ACSMs), a comparatively young and healthy population, from 2005 through 2024.

## Methods

Data for this study were obtained from the Defense Medical Surveillance System (DMSS), a relational database that documents military and medical data for U.S. service members throughout their military careers. Incident cases of oropharyngeal cancer were identified by the presence of a single inpatient encounter with a qualifying diagnosis in the first diagnostic position (International Classification of Diseases, 9th Revision, Clinical Modification [ICD-9-CM]: 141.0, 141.5, 141.6, 141.8 141.9, 145.3-145.5, 146.0-146.2, 146.3-146.9, 149.0, 149.1, 149.8; International Classification of Diseases, 10th Revision, Clinical Modification [ICD-10-CM]: C01, C02.4, C02.8, C02.9, C05.1, C05.2, C05.8, C05.9, C09.0, C09.1, C09.9, C09.9, C10*, C14.0, C14.2, C14.8), or a ‘V’- or ‘Z’-treatment code (ICD-10-CM: Z51.0, Z51.1, Z51.11, Z51.12; ICD-9-CM: V58.0, V58.1, V58.11, V58.12) in the first diagnostic position and a qualifying diagnosis in the second diagnostic position, or with 3 or more outpatient encounters in a 90-day period with a qualifying diagnosis in the first or second diagnostic position. An individual was counted as an incident case only once per lifetime. Person-time was counted in years of active component service and was censored at the date of incident diagnosis. Multivariable Poisson regression models were used to calculate adjusted incidence rate ratios for service branch, rank, and military occupation, after controlling for age, sex, and racial and ethnic group.

## Results

From 2005 through 2024, 341 new cases of malignant oropharyngeal cancer were diagnosed among U.S. ACSMs, corresponding to an incidence rate (IR) of 1.27 cases per 100,000 person-years (p-yrs) **(Table 1)** . There was no clear increase or decrease in annual incidence observed during the surveillance period **(Figure)** . Instead, IRs fluctuated between a low of 0.79 cases per 100,000 p-yrs in 2023 and high of 1.87 cases per 100,000 p-yrs in 2014. The most common anatomical site of incident diagnosis was the tonsil (n=94, 28%), followed by other and unspecified parts of the tongue (n=85, 25%), oropharynx (n=76, 22%), base of the tongue (n=54, 16%), illdefined sites of lip, oral cavity and pharynx (n=20, 6%), and soft palate (n=12, 3.5%) (data not shown).

**TABLE 1.** Incident Cases and Rates of Diagnoses for Malignant Oropharyngeal Cancer, U.S. Active Component Service Members, 2005–2024

	No.	Rate ^a^
Total	341	1.3
Sex		
Male	331	1.5
Female	10	0.2
Branch of service		
Army	160	1.6
Navy	76	1.2
Air Force, Space Force	88	1.3
Marine Corps	17	0.5
Race and ethnicity		
White, non-Hispanic	254	1.6
Black, non-Hispanic	27	0.6
Hispanic	26	0.7
Other	25	1.0
Unknown	9	1.7
Age, *y*		
17–24	17	0.2
25–29	23	0.4
30–34	37	0.9
35–39	56	1.8
40–44	94	5.3
45–49	61	8.7
50–54	34	16.1
55–59	15	30.6
60+	4	46.4
Military rank		
Enlisted	209	0.9
Officer	132	2.9
Military occupation	.	.
Combat-related	51	1.3
Motor transport	11	1.4
Pilot, air crew	20	2.0
Repair, engineering	85	1.1
Communications, intelligence	63	1.1
Health care	43	1.9
Other	68	1.3

Abbreviations: No., number; *y* , years.

a Rates per 100,000 person-years.

**FIGURE:**
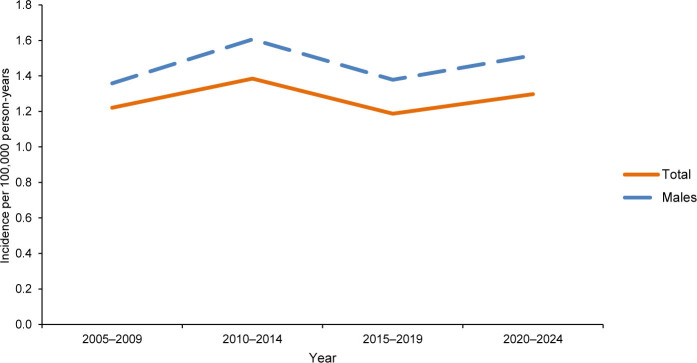
Incidence Rates of Oropharyngeal Cancer, U.S. Active Component Service Members, 2005–2024

Incidence of oropharyngeal cancer was 6 times higher in male ACSMs compared to female ACSMs, with rates increasing significantly with increasing age **(Table 1)** . Non-Hispanic White ACSMs had the highest rate, compared to the other known racial and ethnic groups. Compared to other service branches, ACSMs in the Marine Corps had the lowest rate, and Army members had the highest rate, which remained true even after adjustment for age, sex, and race and ethnicity **(Table 2)** . Officers had a higher crude incidence compared to enlisted members; however, this was no longer true in the adjusted analysis. Pilots and air crew had the highest crude IRs compared to other occupations, but ACSMs in motor transport occupations had the highest adjusted rates.

**TABLE 2.** Crude and Adjusted Rate Ratios for Oropharyngeal Cancer, U.S. Active Component Service Members, 2005–2024

	Crude	Adjusted ^a^	
Branch of service	IRR	IRR	*p* -value
Army	Reference	Reference	Reference
Navy	0.7	0.7	0.0
Air Force, Space Force	0.8	0.8	0.2
Marine Corps	0.3	0.5	<0.01
Military rank			
Enlisted	Reference	Reference	Reference
Officer	3.0	0.9	0.2
Military occupation			
Combat-related	0.7	1.4	0.2
Motor transport	0.7	2.2	0.0
Pilot, air crew	Reference	Reference	Reference
Repair, engineering	0.5	1.2	0.4
Communications, intelligence	0.5	1.1	0.7
Health care	0.9	1.3	0.3
Other	0.6	1.2	0.4

Abbreviation: IRR, incidence rate ratio.

a Adjusted for age category, sex, and racial and ethnic group.

## Discussion

The demographic and time trends of oropharyngeal cancer incidence among ACSMs observed in this analysis are similar to the findings from a 2021 *MSMR* report on oral cavity and pharynx cancers, with men and older service members showing higher rates of diagnosis. ^2^ Unlike trends observed in the U.S. during a similar period, annual IRs in ACSMs did not increase over time—but there was no obvious decrease. Overall oropharyngeal cancer incidence in the U.S. is not expected to be affected significantly by the HPV vaccine until 2045, as older individuals who did not receive an HPV vaccine will remain at increased risk until then. ^6 , 9^

In the U.S., oropharyngeal cancer rates are slightly higher among non-Hispanic White individuals compared to other racial and ethnic groups, which was consistent with this report's findings among ACSMs. ^10 , 11^ One hypothesized reason for this trend includes varying oral sexual behaviors, which are associated with high-risk HPV infection, among different racial and ethnic groups. ^12^ Other potential hypotheses include birth cohort effects, varied levels of smoking behaviors, as well as socio-economic factors and access to health care. ^12^ This study did not intend to compare oropharyngeal incidence rates to the U.S. population. It would not be appropriate to use the findings of this study to compare to the U.S. population due to differences in case ascertainment methodology and underlying population differences. Instead, the intent of this study was to evaluate internal trends of oropharyngeal cancer within the active component U.S. military. Limitations of this study included the fact that annual incidence trends could not be evaluated among subgroups by sex or age due to the small number of cases identified during the long surveillance period. In addition, data on risk and protective factors including alcohol use, smoking, and full HPV vaccination history were not available.

Oropharyngeal cancer is rare among ACSMs, likely due to the fact it is a cancer primarily affecting older age groups, with an average age of onset age 64 years. ^13^ Because 20% of oropharyngeal cancers are estimated to occur in individuals younger than age 55 years, however, continued surveillance of population cancer rates is recommended to determine the evolving impacts of vaccination and changing lifestyle factors. ^13^ Surveillance of cancer trends is necessary for maintaining a fit and medically ready military fighting force, ensuring long-term operational effectiveness, and helping to identify service-related environmental trends or risk factors.
